# CardioBengo study protocol: a population based cardiovascular longitudinal study in Bengo Province, Angola

**DOI:** 10.1186/s12889-016-2759-9

**Published:** 2016-03-01

**Authors:** João M. Pedro, Edite Rosário, Miguel Brito, Henrique Barros

**Affiliations:** Health Research Centre of Angola (CISA), Caxito, Angola; EPIUnit, Institute of Public Health, University of Porto, Porto, Portugal; Lisbon School of Health Technology, Lisbon, Portugal; Department of Clinical Epidemiology, Predictive Medicine and Public Health, University of Porto Medical School, Porto, Portugal

**Keywords:** Cardiovascular risk factors, Incidence, Prevalence, Epidemiology, Angola, Sub-Saharan Africa

## Abstract

**Background:**

Cardiovascular diseases and other non-communicable diseases are major causes of morbidity and mortality, responsible for 38 million deaths in 2012, 75 % occurring in low- and middle-income countries. Most of these countries are facing a period of epidemiological transition, being confronted with an increased burden of non-communicable diseases, which challenge health systems mainly designed to deal with infectious diseases. With the adoption of the World Health Organization “Global Action Plan for the Prevention and Control of non-communicable diseases, 2013–2020”, the national dimension of risk factors for non-communicable diseases must be reported on a regular basis. Angola has no national surveillance system for non-communicable diseases, and periodic population-based studies can help to overcome this lack of information. CardioBengo will collect information on risk factors, awareness rates and prevalence of symptoms relevant to cardiovascular diseases, to assist decision makers in the implementation of prevention and treatment policies and programs.

**Methods:**

CardioBengo is designed as a research structure that comprises a cross-sectional component, providing baseline information and the assembling of a cohort to follow-up the dynamics of cardiovascular diseases risk factors in the catchment area of the Dande Health and Demographic Surveillance System of the Health Research Centre of Angola, in Bengo Province, Angola. The World Health Organization STEPwise approach to surveillance questionnaires and procedures will be used to collect information on a representative sex-age stratified sample, aged between 15 and 64 years old.

**Discussion:**

CardioBengo will recruit the first population cohort in Angola designed to evaluate cardiovascular diseases risk factors. Using the structures in place of the Dande Health and Demographic Surveillance System and a reliable methodology that generates comparable results with other regions and countries, this study will constitute a useful tool for the surveillance of cardiovascular diseases. Like all longitudinal studies, a strong concern exists regarding dropouts, but strategies like regular visits to selected participants and a strong community involvement are in place to minimize these occurrences.

## Background

Cardiovascular diseases (CVDs) are a leading cause of death. In 2012 they were responsible for 17.5 million deaths worldwide, and along with other non-communicable diseases (NCDs) such as cancers, chronic respiratory diseases and diabetes they constitute by far the major causes of death that year. Around 75 % (28 million) of those deaths happened in low- and middle-income (LMI) countries. Of the 16 million of deaths caused by NCDs before the age of 70, 82 % took place in those countries [1].

CVDs and other NCDs share common risk factors such as obesity, hypertension, hyperglycaemia or hypercholesterolemia, smoking, physical inactivity, unhealthy diets or alcohol [1, 2]. NCDs behavioural risk factors are commonly associated with life styles whose consequences usually manifest at older ages in high-income countries. However, in the LMI countries NCDs are rising faster and affecting younger people, therefore presenting earlier those consequences of adverse health and economic outcomes [3, 4].

The growing burden of NCDs with their associated premature mortality, are a major concern for health systems decision-makers, especially in developing countries, were NCDs growth outpaced the reduction in communicable diseases by 33 % between 1990 and 2010 [3]. The “Global Action Plan for the Prevention and Control of NCDs, 2013–2020” adopted by all member states of the World Health Organization (WHO) defined nine voluntary global targets to be achieved by 2025, one being a 25 % reduction from 2010 levels in NCD related preventable deaths (referred to as the 25x25 target) [5]. All signatories have committed to report national levels of NCD risk factors on a regular basis, reinforcing the key recommendations of the 2011 United Nations high-level meeting on NCDs which included the strengthening of surveillance of NCD [6], that allows a global and integrated mechanism to support the implementation of prevention and treatment policies and programs.

The WHO STEPwise approach to Surveillance (STEPS) was designed in 2007 to provide core data on the established risk factors for the major NCDs based on standardized questions and protocols that enable appropriate comparisons across surveys. It uses a modular technique, which allows to fit the specific needs and the available resources for each setting [7].

STEPS was used in 36 African countries, between 2003 and 2013 [8], but not in Angola where four studies conducted between 2008 and 2014 provided the available information on CVDs risk factors using other methodologies: a survey in 667 adult students of Health Sciences in Lubango, south of the country [9]; a study conducted in 615 active employees of a public university in Luanda, the capital of Angola [10]; a survey with 421 subjects from a rural community near Luanda [11]; and a community-based survey with 1464 participants conducted by the Health Research Centre of Angola (CISA) in the catchment area of the Dande Health and Demographic Surveillance System (Dande-HDSS) [12].

Using the Dande-HDSS structure for data collection and management setting, we designed a new study, named CardioBengo, to address the surveillance of the major CVD risk factors with special emphasis on the agreed selected NCD risk factors: tobacco use, harmful alcohol use, salt intake, obesity, raised blood pressure, raised blood glucose and diabetes and physical inactivity but also covering the additional targets that focus on treating people at high risk of heart attack and stroke and on the availability of drugs to treat NCDs. Accordingly, the study aimed to:

- Quantify the prevalence, awareness, treatment and control of hypertension, hyperglycaemia and hypercholesterolemia and their socio-demographic and behavioural determinants, as a baseline for future assessments;
- Describe tobacco, alcohol consumption, physical activity and diet patterns;
- To run follow-up assessments every 3 years, to monitor and understand trends in risk factors and outcomes frequency.

## Methods

### Study design and setting

CardioBengo is a research to be conducted in the catchment area of the Dande-HDSS, located in the Dande Municipality, Bengo Province, 60 km north of Luanda, the capital of Angola. Dande-HDSS monitors the structure, dynamics and geographical distribution of a population of 60,075 people (53.5 % adults) that spreads across an area of 4700 km^2^ with 70 hamlets across three communes (Caxito, Mabubas and Úcua) [13]. This HDSS constitutes the base for the identification of a representative population sample to be invited to participate in the cohort providing a baseline assessment and a longitudinal follow-up evaluation planned at every 3 years, taking the same approach used at baseline.

### Sample size estimation and sampling

The sample size is calculated assuming a simple random sample, based on the 23 % prevalence of hypertension found for this population in a study conducted in 2011 [12], and anticipating a response rate of 70 %. The sample size estimates were generated for males and females in five age groups between 15 and 64 years old, resulting in a total of 3515 individuals. A random list will be drawn from the Dande-HDSS population database.

### Inclusion and exclusion criteria

The inclusion criteria are: i) living in the Dande-HDSS study area at the time of the field work, ii) willingness to participate in this study, and iii) age between 15 and 64 years. The exclusion criteria are: i) subjects with missing anthropometric or blood pressure values and ii) pregnant women; because their biochemical and anthropometric parameters would vary from the non-pregnant physiology they will be excluded from the baseline analysis.

### Pilot study

A pilot study was conducted to test data collection instruments, procedures and to improve the training of field workers. Adjustments in the language used in the questionnaires were made, and the field logistics optimized to a model of five testing protocol stations, namely i) registry and socio-demographic data, ii) point of care clinical tests, iii) anthropometric measures and food habits, iv) blood pressure measurement and drug treatment history and v) electrocardiogram, for the field work facilities. The median time for each participant to pass through all five stations was calculated in 2 h, reaching to the conclusion that a maximum of 25 participants in an 8 h day’s work is possible. All field workers and health professionals selected to collaborate in the study were involved on the pilot study and their skills tested and improved, if necessary.

### Enrolment of participants

One day before the arrival of the team that will implement all the procedures related with the protocol, a field worker visits the hamlets in order to prepare the necessary arrangements for the field work, namely engaging with the local authorities, involving them in the process of selection of an adequate location to install the study facilities and in the door-to-door contact of the prior selected participants, in order to sensitize them to the objectives of the study and to schedule their participation for the next days.

Selected individuals will be consider unreachable after 2 failed attempts of contact in their household at different hours in weekdays, and a refusal will be recorded when the contacted person explicitly says that he or she doesn’t want to participate.

Each participant is expected to spend up to two hours to complete the study tasks, receiving a small lunch at the end, in order to compensate the fasting period previous to the exams. After the exams all participants will receive a card summarizing their clinical and anthropometric results and those with abnormal levels of blood pressure, glucose, cholesterol or other clinical alteration will be referred to the General Hospital of Bengo for a follow-up evaluation with a medical doctor.

### Study procedures

All procedures will be conducted by trained interviewers from the Dande-HDSS and certified health professionals (nurses, laboratory technicians and cardiopneumographic technician) able to communicate in Portuguese (official language) and in the local languages. The protocol for data collection is based on the WHO STEPS manual version 3.0 [7], translated to Portuguese, pre-tested and piloted as referred above. All core items are included and some of the expanded items adapted to the specific context.

### Demographic and social characteristics

Information on age, sex, marital status, current occupation, completed years of school frequency and household income will be obtained using a questionnaire following the STEPS manual [7].

### Behavioural measurements

Information on smoking, namely current smoking status and number of cigarettes smoked, will be collected as described in the STEPS manual [7]. Additionally, a validated Portuguese version of the Fagerström test [14], is used to assess smoking dependency. Alcohol intake, the daily consumption of fruit, vegetables and oil or fat, will be measured according STEPS manual [7]. The optional module for dietary salt is also included in the questionnaire [7]. Physical activity and sedentary behaviour will be assessed using all core and expanded items of the STEPS manual [7].

### Medication history

Following the STEPS guidelines [7], the history of eachindividual regarding hypertension, hyperglycaemia and hypercholesterolemia, is obtained through questions evaluating previous measurements of high blood pressure, glycaemia and blood cholesterol. When any of these items are identified as positive, the participants will be asked about history of recommendations or degree of awareness of such conditions transmitted by a health professional. Any individual will be considered under treatment if he/she indicates the use of specific drug therapy and considered controlled if he/she presents a normal measurement under a specific drug therapy.

### Female reproductive history

Data will be collected on menarche, reproductive history (number of pregnancies and outcomes), pregnancy induced hypertension, menopause and contraception methods used.

### Clinical and anthropometric assessments

Physical exams will be performed with individuals wearing light clothing, with no footwear and after an overnight fast. For all participants, fasting conditions (more then eight hours without eating) will be recorded. Waist and hip circumferences will be measured to the nearest 0.1 cm using circumference tape SECA 203 (SECA United Kingdom, Birmingham, UK). Body weight will be measured to the nearest 0.1 kg using a digital scale SECA 803 (SECA United Kingdom, Birmingham, UK). Height will be measured to the nearest 0.1 cm in the standing position using a portable stadiometer SECA 213 (SECA United Kingdom, Birmingham, UK). Body mass index and waist to hip ratio will be calculated and categorized according WHO guidelines [15, 16]. Blood pressure will be measured using the automatic sphygmomanometer OMRON M6 Comfort (OMRON Healthcare Europe B.V., Hoofddorp, The Netherlands), as recommended [17]. Each participant will rest for 15 min before measurements: seated, at the right arm, with an appropriate cuff size and 3 readings done at 3 min intervals. An electrocardiogram and rhythm strip will be recorded from all participants using a 12-channel electrocardiograph AsCARD Mr.Grey V 201 (ASPEL, Zabierzów, Poland), that will be recorded digitally using the data base software CARDIO TEKA v001 (ASPEL, Zabierzów, Poland). Respecting the right to privacy of the participants, the electrocardiogram will be conducted in the most private manner behind close curtains.

### Biological sample collection

Blood sugar will be measured using a blood glucose meter ACCU-CHEK Aviva (Roche Diagnostic, Indianapolis, IN, USA) with ACCU-CHEK AVIVA Glucose reactive strips (Roche Diagnostic, Indianapolis, IN, USA). Total cholesterol on the blood will be measured only for participants with more then eight hours fasting, using a point-of-care device ACCUTREND Plus (Roche Diagnostic, Indianapolis, IN, USA) with ACCUTREND CHOLESTEROL reactive strips (Roche Diagnostic, Indianapolis, IN, USA). A urine sample will be collected for assessment of urinary creatinine and microalbumin using test strips AUTION SCREEN (Arkray Europe, Amstelveen, The Netherlands). Finger-prick blood samples will be collected and spotted onto Whatman™ 3MMChr filter paper (Fisher Scientific, Pittsburgh, PA, USA). Blood spots on filter paper will be left to air dry and them stored at 4 °C.

### Referral of participants with clinical alterations

All participants with abnormal clinical results will be referred to the General Hospital of Bengo for a medical visit. A specific form was designed to follow clinical characteristics and treatment prescriptions. The adherence to treatment is evaluated according Morisky Medication Adherence Scale of 4 items [18].

### Future study waves

It’s expected to follow-up the cohort every 3 years using the same protocol, enabling the identification of trends and associations between risk factors and outcomes.

Using the Verbal Autopsy System of the Dande-HDSS adapted from the WHO standardized form [19], data will be crossed and deaths of individuals in this cohort can be identified and linked. For the follow-up of the cohort and aiming to minimise losses, the tracking of participants will be based on the periodic visits to all households of the study area held in the scope of the Dande-HDSS.

### Quality control

The training of the field workers was conducted until the required competency was achieved for their specific tasks, and tested on the pilot study. A senior staff member of Dande-HDSS, responsible for the participant’s enrolment and process trough the survey, will supervise all the fieldwork. Participants will be taken through the testing protocol in a sequential method, to prevent loss of data or stack in a particular station. Raw data from the field will be supervised every week to ensure the quality of data collected. Regular feedbacks between the field team and research assistants are schedule. All equipment will be maintained, serviced and calibrated according to manufacturer’s specifications and schedules.

### Data management

All questionnaires will be coded by experienced trained personnel and data will be double entered into a PostgreSQL® database. A series of logic checks will be performed and if any outliers are encounter, discrepancies will be followed up with research assistants and field supervisors. All digital data will be stored in password-protected files and hard copies of questionnaires warehoused in locked cabinets in the facilities of CISA, in Angola. Dande-HDSS supervision team will manage the access to the databases, ensuring the confidentiality of personal records of the participants. Files were designed to store all information related to participant identification separated from the remaining data collected so that all procedures, namely analysis, are maintained under anonymity.

### Statistical analysis

Descriptive data will be reported as absolute frequencies and percentages, means and standard deviations as appropriate. Contingency tables with Pearson chi-squared tests and pairwise correlations will be used to identify variables associated with hypertension, hyperglycemia and hypercholesterolemia. Logistic regression models will be fitted, adjusting for potential confounding factors. Crude and adjusted odds ratios should be estimated for the identified variables with significant association. Adjusted prevalence ratios, computed using Poisson regression models with robust estimator [20], will also be used when appropriate. During follow-up, incidence rates will be calculated as the number of new cases divided by the total person-time at risk. Time at risk will be counted as the time between 2 evaluations for subjects who remained free of the index problem, and for the new cases, time at risk will be assumed as the midpoint of the time interval between evaluations. 95 % confidence intervals will be calculated and a significance level of *p* < 0.05 will be set for all determinations.

## Discussion

The rise in CVDs and other NCDs is a major issue for global health, particularly in LMI countries, though not all countries have the resources to build national surveillance systems to provide solid data on the epidemiology of such diseases. In their absence and of the data from a nationwide STEPS, monitoring of NCDs can be envisaged by structures such as the Dande-HDSS, which may contribute for the surveillance of the major chronic NCD risk factors in representative populations [21].

Due to unequal access to health care, periodic population-based studies based in HDSS can help to overcome the lack of health information, being useful in detecting variables such as risk factors, awareness rates and prevalence of symptoms, otherwise difficult to register in a facility-based system [22].

Being in an early stage of an epidemiological transition, Angola presents at the same time, an increase in premature deaths cause by NCDs and high rates of maternal and chid mortality caused by infections diseases [23]. The country stills records one of the highest rates of maternal mortality in the world (460 deaths per 100,000 live births) [24]. In this sense, the inclusion of a female reproductive history in this study and the possibility of cross information with morbidity and mortality data are an important component for the study of maternal health.

As in any longitudinal study, there will be a risk of dropout. Such problem is more common in cohort studies in Sub Saharan countries due to significant migration patterns and absence of usual means of follow-up used in high income-countries, such as central electronic medical records or unique identifiers to link different datasets [22, 25]. However, as described earlier, we predict to have periodic visits to all participants to keep them motivated between surveys, with a strong effort in all contacts to explain the purpose of the study and make people feel they are making a contribution. The engagement of local authorities is a key factor is this process, helping to build a relationship of trust with the participants. Also, additional resources and strategies can be applied to further follow-ups, such as the use of cell phones to contact and schedule follow-up visits and use weekend days to collect data.

Considering the settings where the protocol was applied, often in rural and inhospitable access hamlets, with straw dwellings and precarious structures, it will be necessary to create conditions for the application of the survey, in case of lack of an appropriate local structure (as a school, a church or a health centre). For this matter, the team projected and prepared a kind of a tent, using awnings and screens to ensure the comfort and privacy of the participants in the study and use it as study platform.

These harder conditions of the terrain and in the absence of local facilities to conduct laboratory tests or a reliable chain of refrigeration to maintain the right storage conditions for venous blood and urine, we choose to conduct dry chemistry using point of care devices that insure reliable results. In future follow-up actions, the possibility to adopt other laboratory methodologies is under consideration, such as the inclusion of the determination of the ratio of apolipoprotein B to apolipoprotein A-1.

Even using the STEPS methodology for better comparisons, due to the methodological differences namely in sampling frames and age groups used, direct comparisons between studies sometimes are not possible. The majority of the studies regarding CVDs and their risk factors only include individuals aged above 25 years. Having this in mind, we decided to maintain individuals aged 15–24 years in this study, because we want to create a cohort with a long-term follow-up, detecting how CVDs risk factors evolve in younger fractions of the population. If require to better compare results with other studies the necessary steps will be made to calculate the adjusted prevalence’s.

In summary, CardioBengo can represent a solid baseline to launch a cohort able to provide critical data for the development of meaningful public health policy, integrated in the Angolan National Plan of Sanitary Development for the period 2012–2025, that predicts the need of further knowledge on CVDs in Angola to guide health promotions initiatives to the right group and problem [26].

## Ethics approval and consent to participate

The Ethics Committee of the Angolan Ministry of Health has approved this protocol. In addition the study has received also the approvals of the ethics committee of the Institute of Public Health, University of Porto (ref CE14010), in the context of the doctoral programme in Public Health of JMP. A written informed consent was obtained from each participant (in the case of under 18 years old, their parent or legal guardian). A copy of the signed consent form as well as instructions regarding the fasting period and contact information was delivered to each participant.

## Consent for publication

Not applicable.

## Availability of data and material

All datasets are available from the CISA Data Base for researchers eligible for access upon request to info@cisacaxito.org.

## Abbreviations

CISA: Health Research Centre of Angola
CVDs: cardiovascular diseases
Dande-HDSS: Dande Health and Demographic Surveillance System
LMI: low- and middle-income
NCDs: non-communicable diseases
STEPS: STEPwise approach to Surveillance
WHO: World Health Organization
